# Effects of β-glucan with vitamin E supplementation on the growth performance, blood profiles, immune response, pork quality, pork flavor, and economic benefit in growing and finishing pigs

**DOI:** 10.5713/ab.22.0433

**Published:** 2023-02-26

**Authors:** Tae Wook Goh, Hong Jun Kim, Kunyong Moon, Yoo Yong Kim

**Affiliations:** 1 Department of Agricultural Biotechnology, and Research Institute of Agriculture and Life Sciences, Seoul National University, Seoul 08826, Korea

**Keywords:** β-Glucan, Economic Benefit, Growing and Finishing Pig, Growth Performance, Vitamin E

## Abstract

**Objective:**

This study was conducted to evaluate the effects of β-glucan with vitamin E supplementation on the growth performance, blood profiles, immune response, pork quality, pork flavor, and economic benefit in growing and finishing pigs.

**Methods:**

A total of 140 growing pigs ([Yorkshire×Landrace]×Duroc) were assigned to five treatments considering sex and initial body weight (BW) in 4 replications with 7 pigs per pen in a randomized complete block design. The experimental diets included a corn-soybean meal-based basal diet with or without 0.05% or 0.1% β-glucan and 0.02% vitamin E. The pigs were fed the diets for 12 weeks (phase I, 0 to 3; phase II, 3 to 6; phase III, 6 to 9; phase IV, 9 to 12). The BW and feed intake were measured at the end of each phase. Blood samples were collected at the end of each phase. Four pigs from each treatment were selected and slaughtered for meat quality. Economic benefit was calculated considering the total feed intake and feed price. Pork flavor was analyzed through inosine monophosphate analysis.

**Results:**

The average daily gain and feed efficiency were improved compared to the control when β-glucan or vitamin E was added. Supplementing 0.05% β-glucan significantly increased the lymphocyte concentration compared to the addition of 0.1% β-glucan and the content of vitamin E in the blood increased when 0.02% vitamin E was added. The treatment with 0.1% β-glucan and 0.02% vitamin E showed the most economic effect because it had the shortest days to market weight and the lowest total feed cost. The addition of β-glucan or vitamin E had a positive role in improving the flavor of pork when considering that the content of inosine monophosphate was increased. However, carcass traits and meat quality were not affected by β-glucan or vitamin E.

**Conclusion:**

The addition of 0.1% β-glucan with 0.02% vitamin E in growing and finishing pig diets showed great growth performance and economic effects by supplying vitamin E efficiently and by improving the health condition of pigs due to β-glucan.

## INTRODUCTION

Antibiotics have been widely used to improve growth performance and prevent disease in livestock environments for a long time. However, the use of antibiotics is harmful to animal and human and causes serious problems, such as bacterial resistance to antibiotics, presence of drug residues in pork products, and environmental pollution [1]. Therefore, the European Union and Korea banned the use of antibiotics as feed additives for growth promotion in 2006 and 2011, respectively. For this reason, research on various antibiotic substitutes, such as plant extracts, probiotics, and β-glucan, has been actively conducted [2].

β-Glucan is a type of functional polysaccharide and is a component contained in mushrooms, grain seeds (oat, rye, barley, etc.), and the cell walls of yeast. It has various biological functions, such as improving immune function, preventing disease, and controlling blood glucose [3]. β-Glucan can stimulate a series of pathways that strengthen the immune system and enhance both innate and acquired immunity [4]. An active immune system can help animals fight disease-causing organisms and maintain clinical infection control and growth processes. In addition, β-glucan has beneficial effects on improving growth performance, nutrient digestibility, and carcass traits when it is added to growing and finishing pig feed [5].

Vitamin E, which acts as an antioxidant at the cell membrane level, has a structural function and performs several functions related to reproduction [6]. Adding a high vitamin E content in the growing and finishing pig feed increases the immune response [7]. Vitamin E can prevent oxidized phospholipids in cell membranes and fats in feed, including unsaturated fatty acids [8]. In addition, vitamin E mainly maintains cells and helps to minimize the damage caused by oxidative reactions in cells [9].

There have been many previous studies evaluating the effects of β-glucan with vitamin E on growing and finishing pigs, but there is insufficient evidence to verify the synergistic effect of β-glucan with vitamin E on growing and finishing pigs. Therefore, this study was conducted to evaluate the effects of β-glucan with vitamin E on the growth performance, blood profiles, immune response, pork quality, pork flavor, and economic benefit in growing and finishing pigs.

## MATERIALS AND METHODS

All experimental procedures involving animals were conducted in accordance with the Animal Experimental Guidelines provided by the Seoul National University Institutional Animal Care and Use Committee (SNUIACUC; SNU-200209-2)

### Experimental animals and management

A total of 140 growing pigs ([Yorkshire×Landrace]×Duroc) with an initial body weight (BW) of 34.43±2.362 kg were assigned to one of five treatments considering sex and initial BW in four replicates with seven pigs per pen in a randomized complete block design. Pigs were randomly allotted to their respective treatments by the experimental animal allotment program (EAAP) [10]. Pigs were housed in an environmentally controlled facility. The pens were fully concrete floored (2.60×2.84 m^2^) and equipped with a feeder and water nipple. The experimental period was 12 weeks (phase I, 0 to 3; phase II, 3 to 6; phase III, 6 to 9; phase IV, 9 to 12).

### Experimental design and diet

Dietary treatments included i) CON, corn–soybean meal (SBM)-based diet; ii) LB, corn-SBM-based diet + 0.05% β-glucan; iii) LBE, corn-SBM-based diet + 0.05% β-glucan + 0.02% vitamin E; iv) HB, corn-SBM-based diet + 0.1% β-glucan; and v) HBE, corn-SBM-based diet + 0.1% β-glucan + 0.02% vitamin E. A corn-SBM-based diet was used as feed in this experiment, and all nutrients in the experimental diet except crude protein (CP) met or exceeded the nutrient requirements of the National Research Council (NRC) [11] for growing and finishing pigs. The CP was set to 6.25 times more than the standard total nitrogen in the requirement of NRC [12]. In the present study, β-glucan and vitamin E products were provided by E&T Company (E&T CO., Ltd, Daejeon, Korea). β-Glucan consisted of (1,3)-(1,6)-β-D-glucan and mannan, and vitamin E was in the form of vitamin E-acetate. In the case of vitamin E, 35 IU/kg was present in the vitamin premix, and 110 IU/kg of vitamin E was additionally supplemented to the LBE and HBE treatments. All nutrient contents in the feed were formulated equally, and the formula and chemical composition of the experimental diet are presented in Table 1 to 4.

### Growth performance

Body weight and feed intake were measured at the end of each phase to calculate the average daily gain (ADG), average daily feed intake (ADFI), and gain:feed ratio (G:F ratio). In addition, feed given to all growing and finishing pigs was recorded each day, and feed waste in the feeder was recorded at the end of each phase.

### Blood profiles and immune response

Blood samples were taken from the jugular veins of three pigs near the average BW in each treatment after 3 hours of fasting on the initial day, week 3, 6, 9, and 12 to measure vitamin E, selenium (Se), tumor necrosis factor-α (TNF-α), interleukin-6 (IL-6), and lymphocytes. All blood samples were collected in serum tubes (SST II Advance; BD Vacutainer, Becton Dickinson, Plymouth, UK) and centrifuged at 1,957×g and 4°C for 15 min (5810R; Eppendorf, centrifuge 5810R, Hamburg, Germany). Subsequently, the supernatant was separated in a microtube (AXYGEN. INC, Union City, CA, USA), and the samples of vitamin E, IL-6, and TNF-α were stored at −20°C, while the samples of Se and lymphocytes were stored at 4°C for analysis. Each measurement was conducted using the following analysis machines and techniques: Se (inductively coupled plasma–mass spectrometry (ICP–MS); Perkin Elmer, Rodgau, Germany), vitamin E (high-performance liquid chromatography [HPLC], HPLC-UVD, PerkinElmer, Milford, MA, USA), lymphocytes (Flow cytometry, automatic blood analyzer; Sysmex, Hyogo, Japan), TNF-α (Fluorescent, Luminex; Millipore, Austin, TX, USA), and IL-6 (Fluorescent, Luminex, Millipore, USA).

### pH and pork color

At the end of the experiment, four finishing pigs from each treatment were selected and slaughtered for pork quality analysis. Pork samples were collected from the nearby 10th rib on the right side of the carcass. Because of the chilling procedure, 30 min after slaughter was regarded as the initial time. The pH and pork color were measured at 0, 3, 6, 12, and 24 h, respectively. The pH was measured using a pH meter (Model 720; Thermo, Orion, Waltham, MA, USA) and pork color was determined by CIE color L*, a*, and b* values using a CM-M6 (Minolta Camera Co., Tokyo, Japan).

### Carcass traits and physiochemical properties

The pig carcass grading was judged using the data determined to be grade 5 according to the criterion for grades for pork carcass (scalding) after slaughter at the slaughterhouse. Only results judged to be grades 1+, 1, 2, and 3 were used, and outside grades (grade E) were not included in the grade. The carcass weight was measured by the hot carcass weight of the half carcass. For the back fat thickness, the thickness of the fat layer was measured by cutting the last rib of the carcass, which was precooled to 5 degrees or less at a right angle. Water holding capacity (WHC) was measured by the centrifuge method [13]. Longissimus muscles were ground and sampled in filter tubes, heated in a water bath at 80°C for 20 min and centrifuged for 10 min at 2,000 rpm and 4°C (Eppendorf centrifuge 5810R, Germany). After that, to calculate the cooking loss, longissimus muscles were packed with polyethylene bags, heated in a water bath until the core temperature reached 70°C and weighed before and after cooking. After heating, the samples were cored (0.5 inch diameter) parallel to muscle fiber and the cores were used to measure the shear force using a Warner-Bratzler meat shearing machine (Salter 235; GR, USA). The shear force, cooking loss and WHC of pork were analyzed by animal origin food science, Seoul National University.

### Pork flavor

For analysis of inosine monophosphate (IMP), which is an indicator to infer the pork flavor, the samples were thawed and centrifuged at 10,000 rpm for 5 min at 4°C (Eppendorf centrifuge 5417R), and the supernatants were transferred to cold HPLC vials and placed in a thermostatted autosampler (1°C to 2°C). Analysis of IMP was performed by high-performance liquid chromatography (HPLC) (Hewlett-Packard HPLC system series 1100; Waldbronn, Germany) using UV detection (210 nm).

### Economic benefit

As the experimental pigs were reared in the same environmental conditions, economic efficiency was calculated using only the feed cost without considering other factors. The total feed cost and feed cost (won) per body weight gain (kg) were calculated using the amount of the total feed intake and the feed price. The days to reach market weight (115 kg) were estimated from the BW at the end of the feeding trial and ADG of 10 to 11 weeks.

### Statistical analysis

All obtained data were first processed by Excel 2010, and then analyzed by one-way analysis of variance using Statistical Analysis System 9.4 TS1M7 (SAS Inst. Inc., Cary, NC, USA). Each pen was used as an experimental unit for growth performance and economic benefit, while individual pigs were used as experimental units for blood profiles, immune response, and pork quality. Orthogonal polynomial contrasts were used to determine the effects of diet (β-glucan and vitamin E against the control), β-glucan, vitamin E, and the interaction between β-glucan and vitamin E. Data are presented as the means and their pooled standard errors. The differences were considered statistically significant when p<0.05, while 0.05≤p<0.10 was considered to indicate a trend in the data.

## RESULTS AND DISCUSSION

### Growth performance

The effects of β-glucan with vitamin E supplementation in the growing and finishing pig diets on growth performance are presented in Table 5. As a result of the experiment, ADG was significantly higher in the early finishing period (Diet, p<0.01) and showed a higher trend in the total finishing period (Diet, p = 0.06) in the treatments in which β-glucan or vitamin E was added compared to the control. A significant difference in the late finishing period (BG×VE, p<0.05) was found in ADFI by the interaction between β-glucan and vitamin E. The G:F ratio was significantly higher in the early finishing period (Diet, p<0.05) and showed a higher trend in the total finishing period (Diet, p = 0.05) when β-glucan or vitamin E was added compared to the control. The treatments supplemented with 0.02% vitamin E showed a lower trend in the early growing period (VE, p = 0.05) but a higher trend in the early finishing period (VE, p = 0.07) compared to the treatments without additional vitamin E. In addition, a significant difference in the late finishing period (BG×VE, p<0.05) and a trend in the total finishing period (BG×VE, p = 0.09) were observed in the G:F ratio by the interaction between β-glucan and vitamin E.

In a previous study by Tran et al [14], supplementation with 0.2% β-glucan increased ADG significantly in the growing, finishing, and growing-finishing periods compared to the control when 0.2% β-glucan or 0.4% vitamin E was added to growing and finishing pig feed (p<0.05). In addition, the feed conversion ratio was significantly lower in the treatment where 0.2% β-glucan was added compared to the control (p<0.05). Cueno et al [15] studied the effects on growth performance and carcass traits when β-glucan was added to the feed from weaning pigs to growing and finishing pigs. Weaning pigs were assigned to one of four treatments considering sex and initial BW in a 2×2 factorial design with two levels of carbadox supplementation (0%, 0.25%) and two levels of a product containing β-glucan (0%, 0.2%). As a result, the final body weight tended to be higher in the treatments to which 0.2% β-glucan was added (BG, p = 0.09). According to Luo et al [5], 0.01% β-glucan significantly increased ADG and improved the G:F ratio in 50 to 75 kg and 25 to 110 kg sections (p<0.05) when β-glucan was added by level (0.005%, 0.01%, 0.02%) to the growing and finishing pig feed. In addition, supplementation with 0.01% β-glucan significantly increased the ADG and G:F ratio in the 75 to 110 kg section compared to the control (p<0.05). Luo et al [5] explained that this was because the digestibility of dry matter, total energy, and CP significantly increased with the addition of 0.01% β-glucan, but the exact mechanism for the improvement in growth performance of growing and finishing pigs was not known [16]. However, β-glucan could have a positive effect on growth performance as a result of improving the intestinal microflora of pigs and promoting the absorption of nutrients in the intestine through improved immunity when β-glucan, an immune enhancer, was added to the feed [16]. There was no significant difference in the growth performance of growing and finishing pigs when vitamin E was added to the growing and finishing pig feed [17,18]. However, Hasty et al [19] reported that the ADFI of finishing pigs increased linearly when β-glucan was added by level (0.005%, 0.01%, 0.02%) to the growing and finishing pig feed. Considering previous studies, it is considered that ADG and feed efficiency of the treatment with β-glucan or vitamin E were higher than those of the control because the immunity and health status of the growing and finishing pigs improved.

Consequently, the addition of β-glucan or vitamin E to the growing and finishing pig feed had a positive effect on the growth performance compared to the control.

### Blood profiles and immune response

The effects of β-glucan with vitamin E supplementation in the growing and finishing pig diet on blood profiles and immune response are presented in Table 6. The concentration of vitamin E in the blood was significantly higher in the LBE and HBE treatments supplemented with 0.02% vitamin E (VE, p<0.01) at the 3rd and 12th weeks and showed higher trends at the 6th and 9th weeks (VE, p = 0.07; 0.09). In addition, the concentration of vitamin E in the blood was significantly higher in the treatment where β-glucan and vitamin E were added at the 3rd and 9th weeks compared to the control (Diet, p<0.05), and showed a higher trend at the 6th week (Diet, p = 0.07). In addition, the TNF-α concentration showed a lower trend in the treatments to which β-glucan or vitamin E was added compared to the control at week 9 (Diet, p = 0.07). Supplementation with 0.05% β-glucan was significantly higher than the treatment with 0.1% β-glucan at 9 and 12 weeks in lymphocyte concentration (Diet, p<0.01). There was no significant difference between treatments in selenium and IL-6 concentrations.

In the present experiment, the lymphocyte concentration was increased when 0.05% β-glucan was added to the feed of growing and finishing pigs. Lymphocytes are part of the adaptive immune system, comprising 20% to 40% of the white blood cell count [20]. Hahn et al [21] conducted an experiment by dividing treatments into 4 groups (control, 0.02% β-glucan, antibiotics, and 0.02% β-glucan + antibiotics) to investigate the effect of β-glucan on immunity in weaning pigs. As a result, a subset of the pig lymphocyte population, major histocompatibility complex-II (week 4), CD-4, and CD-8 (week 8) were found to be higher in the diet supplemented with β-glucan compared to weaning pigs fed other diets. According to Kim et al [22], there was no significant difference between treatments on Day 0 before *Escherichia coli* inoculation when β-glucan was added at each level (0.0054%, 0.0108%) in weaning pig feed. However, the ratio of CD4+ T cells was significantly increased in the treatment where 0.0054% β-glucan was added after 2 and 5 days of *E. coli* inoculation compared to the control. On the other hand, in the experiments of Lee et al [23] and Mao et al [24], there was no significant difference in lymphocytes even when β-glucan was added to the weaning pig feed. Regarding the different effects of β-glucan addition on lymphocytes, Bohn and Bemiller [25] suggested that it may be due to differences in molecular weight, branching degrees, morphology, and intermolecular linkages that can affect the bioactive activity of β-glucan.

In the present experiment, adding 0.02% vitamin E to growing and finishing pig diets increased the concentration of vitamin E in the blood. In particular, these results are important for growing and finishing pigs. The reason is that the main cause of pork quality deterioration is lipid oxidation, which also increases the occurrence of oxidative rancidity and unpleasant odor. These changes eventually shorten the shelf life of pork. Therefore, antioxidants such as vitamin E are often used to reduce the oxidation of pork and improve shelf life and quality. The results of the present experiment suggest that vitamin E was additionally well delivered to growing and finishing pigs, and it can be expected to have a positive effect on the oxidation and quality of pork in finishing pigs in the future.

In conclusion, the addition of 0.05% β-glucan to the growing and finishing pig feed increased the lymphocyte concentration, and the addition of 0.02% vitamin E increased the vitamin E content in the blood.

### pH of pork

The effects of β-glucan with vitamin E supplementation in the growing and finishing pig diets on the pH of pork are presented in Table 7. As a result, the pH of pork was significantly higher in the LBE and HBE treatments with the addition of 0.02% vitamin E than in the treatments without vitamin E treatment 3 hours after slaughter (VE, p<0.05). In addition, treatments with β-glucan and vitamin E were significantly lower than the control (Diet, p<0.01). The treatment with β-glucan and vitamin E was significantly higher than the control 24 hours after slaughter (Diet, p<0.05). In addition, there was a significant difference in the pH of pork 24 hours after slaughter due to the interaction of β-glucan and vitamin E (BG×VE, p = 0.09).

The change in the pH of pork after slaughter is an important factor in determining the quality of pork and affects the freshness, WHC, softness, color, and storage of the pork [26]. Additionally, Park et al [27] reported that the lower the postmortem pH is, the higher the protein content. Additionally, as the pH increased, cooking loss and drip loss decreased, and the WHC increased. The initial pH and final pH after slaughter are used as standards in judging the quality of pork. The initial pH is the predicted value of pale, soft, exudative (PSE) meat, and the final pH is the predicted value of dry, firm, dark (DFD) meat. When blood supply to the muscle is stopped after death, lactic acid production increases due to the anaerobic glycolysis of glycogen stored in the muscle, and the pH of the muscle decreases. This decrease in pH is affected by the handling conditions before and after slaughter, the genetic capacity of the individual, and the rate of anaerobic glycolysis.

The sudden decrease in the pH of pork promotes the outflow of the juice by modifying the protein structure of the muscle, and the outflow of the juice on the surface scatters light to make the pork look pale, resulting in the generation of PSE meat.

According to Luo et al [5], the pH of pork was significantly higher, and the drip loss could be reduced in the treatment with 0.01% β-glucan than those in the control 45 minutes after slaughter when β-glucan was added by level (0.005%, 0.01%, 0.02%) in the feed for growing and finishing pigs. According to the experiment of He et al [28], the pH of pork was significantly the highest and the drip loss was the lowest 45 minutes after slaughter with a reduced lactic acid content and glycolytic potential in the treatments supplemented with 0.02% β-glucan when β-glucan was added by level (0%, 0.005%, 0.01%, 0.02%, 0.04%) in the feed for growing and finishing pigs. This means that the possibility for pork to develop PSE is low. Additionally, no significant difference in the pH of pork was found when β-glucan was added by level in the feed for growing and finishing pigs [29]. Moreover, no significant difference in the pH of pork was observed between the treatments when vitamin E was supplemented [17–19, 30]. In the present experiment, since the pH was not out of the normal range (pH 5.3 to 6.8), there was no negative effect on pork even though it was affected by β-glucan and vitamin E.

In summary, the addition of β-glucan and vitamin E to the feed of growing and finishing pigs does not negatively affect the pH of pigs after slaughter.

### Color of pork

The effects of β-glucan with vitamin E supplementation in the growing and finishing pig diets on the color of pork are presented in Table 8. As a result of the experiment, there was no significant difference in the color of pork between the treatments.

The first thing consumers see when purchasing pork at a store is the color of the meat. Because of these characteristics, the color of the meat has the greatest influence on consumers’ understanding of the quality of pork and making a purchase decision. The color of pork is an important indicator for evaluating its muscular appearance. It is influenced by several factors, including the rate of postmortem glycolysis, intramuscular fat, pigment level and the oxidation state of the pigment [31].

According to a study by Luo et al [5], treatment with 0.01% β-glucan 45 minutes after slaughter showed significantly higher redness compared to the control (p<0.05) when β-glucan was added by level (0.005%, 0.01%, 0.02%) in the feed for growing and finishing pigs. In addition, the yellowness 45 minutes after slaughter was significantly lower than that of the control (p<0.05) in all the treatments where β-glucan was added. According to an experiment by He et al [28], when β-glucan was added by level (0%, 0.005%, 0.01%, 0.02%, 0.04%) in the feed for finishing pigs, the redness significantly increased (linear, p<0.05), and the whiteness significantly decreased 45 minutes after slaughter (linear, p<0.05). When 0.01% [17], 10 and 210 IU/kg [18], and 0.04% [30] vitamin E were added to growing and finishing pig feed, respectively, there was no significant difference in the color of pork between the treatments.

Bendall and Wismer-pederson [32] reported that an increase in yellowness and a decrease in the redness of pork were the result of a decrease in the freshness of the pork. Since there is no negative effect on the redness and yellowness of the pork when supplemented with β-glucan and vitamin E, it does not affect the freshness of the pork. In addition, Joo et al [33] said that if the L value is 58 or more when 24 hours have elapsed after slaughter, it is PSE pork. However, since all treatments showed an ideal value lower than what Joo et al [33] suggested, it was determined that there was no negative effect on pork in the present experiment.

As a result, the addition of β-glucan and vitamin E to the growing and finishing pig feed does not have a negative effect on the color of pork.

### Carcass traits and physiochemical properties

The effects of β-glucan with vitamin E supplementation in the growing and finishing pig diets on carcass traits and physiochemical properties are presented in Table 9. As a result, there was no significant difference between treatments in carcass traits including carcass weight, carcass yield, grade, and back fat thickness, or in physicochemical properties including cooking loss, shear force, and WHC.

There are standards for pork grading. Carcass weight and back fat thickness are the criteria for the first grade, and appearance, meat quality, and defective items are the secondary grade. In particular, the most important items for grading are carcass weight and back fat thickness, which are the first-grade criteria. Kim and In [34] analyzed the characteristics of carcass traits by classifying them into 5 levels (less than 69 kg; 70 to 80 kg; 81 to 90 kg; 91 to 96 kg; 97 kg or more) according to carcass weight. As a result, back fat thickness increased significantly with increasing carcass weight (p<0.05). In addition, the thickness of back fat was classified into 6 levels (less than 15 mm; 16 to 20 mm; 21 to 25 mm; 26 to 30 mm; 31 to 35 mm; over 36 mm). As a result, the carcass weight became significantly heavier as backfat became thicker (p< 0.05).

Cooking loss, which is generally known to have an inverse correlation with WHC, is an indirect indicator of WHC. Shear force is a mechanical measure of the toughness of meat and is known to be highly related to WHC [35]. WHC is a measure of the moisture in meat according to internal and external environmental changes and is determined by the microstructure of meat or the change in moisture content during shredding. Additionally, it is known to be closely related to changes in the pH of meat. WHC is an important factor in the quality of pork. As the WHC increases, the quality of pork improves, but when the WHC decreases, the shear force gets higher.

According to a study by Luo et al [5], there were no significant differences in carcass weight, back fat, and carcass yield among treatments when β-glucan was added by level (0.005%, 0.01%, 0.02%) in the feed for growing and finishing pigs. There were no significant differences among the treatments in the cooking loss and shear force. The drip loss was rapidly reduced in the treatment where 0.01% β-glucan was added (p<0.05), which meant that the WHC of the muscles could be improved. Sampath et al [29] reported no significant differences in cooking loss and WHC when β-glucan extracted from Saccharomyces cerevisiae was added by level (0%, 0.05%, 0.1%) to the feed for growing and finishing pigs. According to the experiment by He et al [28], there were no significant differences in carcass yield and average back fat thickness when β-glucan was added by level (0%, 0.005%, 0.01%, 0.02%, 0.04%) in the feed for finishing pigs. When 0.02% β-glucan was added to the treatment, the cooking loss was significantly decreased (linear, p<0.05), but there was no significant difference in shear force. According to Li et al [30], when vitamin E (0%, 0.04%) and ferulic acid (0%, 0.01%) were added to the feed for growing and finishing pigs, there were no significant differences in back fat thickness, lean percentage, and shear force by vitamin E. Cannon et al [17] reported that there were no significant differences in carcass yield, back fat thickness, drip loss and cooking loss among treatments when 0.01% vitamin E was added to feed for growing and finishing pigs. Wang et al [18] conducted an experiment to add DDGS (0%, 15%, 30%) and vitamin E (10 and 210 IU/kg) to feed for growing and finishing pigs, but no significant differences were observed in carcass weight, carcass yield, and back fat thickness. In addition, shear force and drip loss were significantly reduced when 210 IU/kg vitamin E was added (p<0.05).

Therefore, the addition of β-glucan and vitamin E to the feed for growing and finishing pigs did not have any effect on carcass characteristics or physicochemical properties.

### Pork flavor

The effects of β-glucan with vitamin E supplementation in the growing and finishing pig diets on pork flavor are presented in Table 10. As a result of the experiment, the treatments with β-glucan and vitamin E had a significantly higher content of IMP than the control (Diet, p<0.05). Additionally, there was a trend in the IMP content (BG×VE, p<0.05) due to the interaction between β-glucan and vitamin E.

IMP is an important indicator to infer the flavor of pork and is particularly related to umami. According to a previous study, supplementing 0.005% and 0.01% β-glucan increased the IMP content in pork when β-glucan was added by level (0.005%, 0.01%. 0.02%) to the growing and finishing pig feed [5]. Usually, after slaughter, the oxygen supply to the muscle tissue is stopped, and the energy supplied by phosphocreatine and glycolysis is used for adenosine triphosphate (ATP) synthesis [36]. As phosphocreatine and glycolysis decrease, ATP synthesis stops and begins to be degraded, which was thought to increase the contents of IMP in the present experiment.

As a consequence, the addition of β-glucan with vitamin E in growing and finishing pig feed had a positive role in improving the flavor of pork when considering that the content of IMP was increased.

### Economic benefit

The effects of β-glucan with vitamin E supplementation in the growing and finishing pig diet on economic benefit are presented in Table 11. In the case of feed cost per weight gain, LBE and HBE treatments with the addition of 0.02% vitamin E showed a high trend in the early growing phase (VE; p = 0.06). Supplementing β-glucan or vitamin E showed significantly lower feed cost per weight gain compared to the control in the early finishing phase (Diet, p<0.05). The treatments supplemented with β-glucan or vitamin E showed significantly lower total feed cost compared to the control in the late finishing phase (Diet, p<0.05). No significant difference was found in days to market weight. However, the treatment HBE with 0.1% β-glucan and 0.02% vitamin E showed the shortest days to market weight among treatments.

Tran et al [14] conducted an experiment on the effect on economic benefit when 0.2% β-glucan and 0.4% vitamin E were added to the growing and finishing feed. As a result, the treatment with 0.2% β-glucan had a higher feed price compared to the control. However, the treatment supplemented with β-glucan showed a better effect of 118.98% based on 100% of the control due to the high ADG and low cost of veterinary medicines.

Consequently, since the HBE treatment with 0.1% β-glucan and 0.02% vitamin E had the shortest days to market weight and the lowest total feed cost, a positive effect on economic efficiency can be expected.

## CONCLUSION

The ADG and feed efficiency were improved compared to the control when β-glucan or vitamin E was added. Supplementing 0.05% β-glucan significantly increased the lymphocyte concentration compared to the addition of 0.1% β-glucan, and the content of vitamin E in the blood increased when 0.02% vitamin E was added. The HBE treatment with 0.1% β-glucan and 0.02% vitamin E showed the most economic effect because it had the shortest days to market weight and the lowest total feed cost. However, carcass traits and meat quality were not affected by β-glucan or vitamin E.

Therefore, the addition of 0.1% β-glucan and 0.02% vitamin E in growing and finishing pig diets showed great growth performance and economic effects by supplying vitamin E efficiently and by improving the health condition of pigs due to β-glucan.

## Figures and Tables

**Table 1:** Formula and chemical compositions of the experimental diets during growing phase 1 (weeks 0 to 3)

Items	Treatment^1)^				

	CON	LB	LBE	HB	HBE
Ingredient (%)					
Ground corn	68.31	68.20	68.16	68.12	68.07
Soybean meal	23.31	23.34	23.34	23.34	23.35
Wheat bran	4.00	4.00	4.00	4.00	4.00
Tallow	1.32	1.35	1.37	1.38	1.40
L-lysine-HCl, 50%	0.07	0.07	0.07	0.07	0.07
DL-methionine, 99%	0.01	0.01	0.01	0.01	0.01
L-threonine, 98.5%	0.01	0.01	0.01	0.01	0.01
L-tryptophan, 20%	0.01	0.01	0.01	0.01	0.01
MDCP	1.44	1.44	1.44	1.44	1.44
Limestone	1.02	1.02	1.02	1.02	1.02
β-glucan^2)^	0.00	0.05	0.05	0.10	0.10
Vitamin E^2)^	0.00	0.00	0.02	0.00	0.02
Vitamin mix^3)^	0.10	0.10	0.10	0.10	0.10
Mineral mix^4)^	0.10	0.10	0.10	0.10	0.10
Salt	0.30	0.30	0.30	0.30	0.30
Sum	100.00	100.00	100.00	100.00	100.00
Chemical composition (%)^5)^					
ME (kcal/kg)	3,265.00	3,265.00	3,265.00	3,265.00	3,265.00
Crude protein (%)	15.69	15.69	15.69	15.69	15.69
Lysine (%)	0.83	0.83	0.83	0.83	0.83
Methionine (%)	0.25	0.25	0.25	0.25	0.25
Cysteine (%)	0.27	0.27	0.27	0.27	0.27
Threonine (%)	0.60	0.60	0.60	0.60	0.60
Tryptophan (%)	0.15	0.15	0.15	0.15	0.15
Calcium (%)	0.66	0.66	0.66	0.66	0.66
Phosphorus (%)	0.56	0.56	0.56	0.56	0.56

MDCP, mono-dicalcium phosphate; ME, metabolizable energy; SBM, soybean meal.

1) CON, corn-SBM-based diet; LB, corn-SBM-based diet+0.05% β-glucan; LBE, corn-SBM-based diet+0.05% β-glucan+0.02% vitamin E; HB, corn-SBM-based diet+0.1% β-glucan; HBE, corn-SBM-based diet+0.1% β-glucan+0.02% vitamin E.

2) β-Glucan and vitamin E products were provided by E&T company (E&T Co, Ltd. Daejeon, Korea).

3) Provided the following quantities of vitamins per kg of complete diet: vitamin A, 16,000 IU; vitamin D_3_, 3,200 IU; vitamin E, 35 IU; vitamin. K_3_, 5 mg; rivoflavin, 6 mg; calcium pantothenic acid, 16 mg; niacin, 32 mg; D–biotin, 128 μg; vitamin B_12_, 20 μg.

4) Provided the following quantities of minerals per kg of complete diet: Fe, 281 mg; Cu, 288 mg; Zn, 143 mg; Mn, 49 mg; I, 0.3 mg; Se, 0.3 mg.

5) Calculated value.

**Table 2:** Formula and chemical compositions of the experimental diets during growing phase 2 (weeks 3 to 6)

Items	Treatment^1)^				

	CON	LB	LBE	HB	HBE
Ingredient (%)					
Ground corn	74.14	74.04	74.00	73.93	73.90
Soybean meal	18.10	18.12	18.12	18.14	18.14
Wheat bran	4.00	4.00	4.00	4.00	4.00
Tallow	0.99	1.02	1.04	1.06	1.07
L-lysine-HCl, 50%	0.01	0.01	0.01	0.01	0.01
DL-methionine, 99%	0.01	0.01	0.01	0.01	0.01
L-threonine, 98.5%	0.01	0.01	0.01	0.01	0.01
L-tryptophan, 20%	0.01	0.01	0.01	0.01	0.01
MDCP	1.30	1.30	1.30	1.30	1.30
Limestone	0.93	0.93	0.93	0.93	0.93
β-glucan^2)^	0.00	0.05	0.05	0.10	0.10
Vitamin E^2)^	0.00	0.00	0.02	0.00	0.02
Vitamin mix^3)^	0.10	0.10	0.10	0.10	0.10
Mineral mix^4)^	0.10	0.10	0.10	0.10	0.10
Salt	0.30	0.30	0.30	0.30	0.30
Sum	100.00	100.00	100.00	100.00	100.00
Chemical composition (%)^5)^					
ME (kcal/kg)	3,265.00	3,265.00	3,265.00	3,265.00	3,265.00
Crude protein (%)	13.75	13.75	13.75	13.75	13.75
Lysine (%)	0.67	0.67	0.67	0.67	0.67
Methionine (%)	0.23	0.23	0.23	0.23	0.23
Cysteine (%)	0.24	0.24	0.24	0.24	0.24
Threonine (%)	0.52	0.52	0.52	0.52	0.52
Tryptophan (%)	0.15	0.15	0.15	0.15	0.15
Calcium (%)	0.59	0.59	0.59	0.59	0.59
Phosphorus (%)	0.52	0.52	0.52	0.52	0.52

MDCP, mono-dicalcium phosphate; ME, metabolizable energy; SBM, soybean meal.

1) CON, corn-SBM-based diet; LB, corn-SBM-based diet+0.05% β-glucan; LBE, corn-SBM-based diet+0.05% β-glucan+0.02% vitamin E; HB, corn-SBM-based diet+0.1% β-glucan; HBE, corn-SBM-based diet+ 0.1% β-glucan+ 0.02% vitamin E.

2) β-Glucan and vitamin E products were provided by E&T company (E&T Co, Ltd. Daejeon, Korea).

3) Provided the following quantities of vitamins per kg of complete diet: vitamin A, 16,000 IU; vitamin D_3_, 3,200 IU; vitamin E, 35 IU; vitamin. K_3_, 5 mg; rivoflavin, 6 mg; calcium pantothenic acid, 16 mg; niacin, 32 mg; D–biotin, 128 μg; vitamin B_12_, 20 μg.

4) Provided the following quantities of minerals per kg of complete diet: Fe, 281 mg; Cu, 288 mg; Zn, 143 mg; Mn, 49 mg; I, 0.3 mg; Se, 0.3 mg.

5) Calculated value.

**Table 3:** Formula and chemical compositions of the experimental diets during the finishing phase 1 (weeks 6 to 9)

Items	Treatment^1)^				

	CON	LB	LBE	HB	HBE
Ingredients (%)					
Ground corn	79.31	79.22	79.18	79.12	79.08
Soybean meal	13.33	13.34	13.34	13.35	13.36
Wheat bran	4.00	4.00	4.00	4.00	4.00
Tallow	0.63	0.66	0.68	0.70	0.71
L-Lysine-HCl, 50%	0.21	0.21	0.21	0.21	0.21
DL-methionine, 99%	0.01	0.01	0.01	0.01	0.01
L-threonine, 98.5%	0.01	0.01	0.01	0.01	0.01
L-tryptophan, 20%	0.01	0.01	0.01	0.01	0.01
MDCP	1.15	1.15	1.15	1.15	1.15
Limestone	0.84	0.84	0.84	0.84	0.84
β-glucan^2)^	0.00	0.05	0.05	0.10	0.10
Vitamin E^2)^	0.00	0.00	0.02	0.00	0.02
Vitamin mix^3)^	0.10	0.10	0.10	0.10	0.10
Mineral mix^4)^	0.10	0.10	0.10	0.10	0.10
Salt	0.30	0.30	0.30	0.30	0.30
Sum	100.00	100.00	100.00	100.00	100.00
Chemical composition (%)^5)^					
ME (kcal/kg)	3,265.00	3,265.00	3,265.00	3,265.00	3,265.00
Crude protein (%)	12.13	12.13	12.13	12.13	12.13
Lysine (%)	0.66	0.66	0.66	0.66	0.66
Methionine (%)	0.21	0.21	0.21	0.21	0.21
Cysteine (%)	0.21	0.21	0.21	0.21	0.21
Threonine (%)	0.46	0.46	0.46	0.46	0.46
Tryptophan (%)	0.12	0.12	0.12	0.12	0.12
Calcium (%)	0.52	0.52	0.52	0.52	0.52
Phosphorus (%)	0.47	0.47	0.47	0.47	0.47

MDCP, mono-dicalcium phosphate; ME, metabolizable energy; SBM, soybean meal.

1) CON, corn-SBM-based diet; LB, corn-SBM-based diet+0.05% β-glucan; LBE, corn-SBM-based diet+0.05% β-glucan+0.02% vitamin E; HB, corn-SBM-based diet+0.1% β-glucan; HBE, corn-SBM-based diet+0.1% β-glucan+0.02% vitamin E.

2) β-Glucan and vitamin E products were provided by E&T company (E&T Co, Ltd. Daejeon, Korea).

3) Provided the following quantities of vitamins per kg of complete diet: vitamin A, 16,000 IU; vitamin D_3_, 3,200 IU; vitamin E, 35 IU; vitamin. K_3_, 5 mg; rivoflavin, 6 mg; calcium pantothenic acid, 16 mg; niacin, 32 mg; D–biotin, 128 μg; vitamin B_12_, 20 μg.

4) Provided the following quantities of minerals per kg of complete diet: Fe, 281 mg; Cu, 288 mg; Zn, 143 mg; Mn, 49 mg; I, 0.3 mg; Se, 0.3 mg.

5) Calculated value.

**Table 4:** Formula and chemical compositions of the experimental diets during finishing phase 2 (weeks 9 to 12)

Items	Treatment^1)^				

	CON	LB	LBE	HB	HBE
Ingredients (%)					
Ground corn	84.41	84.30	84.27	84.20	84.17
Soybean meal	8.73	8.75	8.75	8.77	8.77
Wheat bran	4.00	4.00	4.00	4.00	4.00
Tallow	0.35	0.39	0.40	0.42	0.43
L-Lysine-HCl, 50%	0.17	0.17	0.17	0.17	0.17
DL-methionine, 99%	0.01	0.01	0.01	0.01	0.01
L-threonine, 98.5%	0.01	0.01	0.01	0.01	0.01
L-tryptophan, 20%	0.02	0.02	0.02	0.02	0.02
MDCP	1.05	1.05	1.05	1.05	1.05
Limestone	0.75	0.75	0.75	0.75	0.75
β-glucan^2)^	0.00	0.05	0.05	0.10	0.10
Vitamin E^2)^	0.00	0.00	0.02	0.00	0.02
Vitamin ix^3)^	0.10	0.10	0.10	0.10	0.10
Mineral mix^4)^	0.10	0.10	0.10	0.10	0.10
Salt	0.30	0.30	0.30	0.30	0.30
Sum	100.00	100.00	100.00	100.00	100.00
Chemical composition (%)^5)^					
ME (kcal/kg)	3,265.00	3,265.00	3,265.00	3,265.00	3,265.00
Crude protein (%)	10.43	10.43	10.43	10.43	10.43
Lysine (%)	0.52	0.52	0.52	0.52	0.52
Methionine (%)	0.19	0.19	0.19	0.19	0.19
Cysteine (%)	0.20	0.20	0.20	0.20	0.20
Threonine (%)	0.38	0.38	0.38	0.38	0.38
Tryptophan (%)	0.10	0.10	0.10	0.10	0.10
Calcium (%)	0.46	0.46	0.46	0.46	0.46
Phosphorus (%)	0.43	0.43	0.43	0.43	0.43

MDCP, mono-dicalcium phosphate; ME, metabolizable energy; SBM, soybean meal

1) CON, corn-SBM-based diet; LB, corn-SBM-based diet+0.05% β-glucan; LBE, corn-SBM-based diet+0.05% β-glucan+0.02% vitamin E; HB, corn-SBM-based diet+0.1% β-glucan; HBE, corn-SBM-based diet+0.1% β-glucan+0.02% vitamin E.

2) β-Glucan and vitamin E products were provided by E&T company (E&T Co, Ltd. Daejeon, Korea).

3) Provided the following quantities of vitamins per kg of complete diet: vitamin A, 16,000 IU; vitamin D_3_, 3,200 IU; vitamin E, 35 IU; vitamin. K_3_, 5 mg; rivoflavin, 6 mg; calcium pantothenic acid, 16 mg; niacin, 32 mg; D–biotin, 128 μg; vitamin B_12_, 20 μg.

4) Provided the following quantities of minerals per kg of complete diet: Fe, 281 mg; Cu, 288 mg; Zn, 143 mg; Mn, 49 mg; I, 0.3 mg; Se, 0.3 mg.

5) Calculated value.

**Table 5:** Effects of β-glucan with vitamin E supplementation on growth performance in growing and finishing pigs

Items	Treatment^1)^					SEM	p-value			
	
	CON	LB	LBE	HB	HBE		Diet	BG	VE	BG×VE
Body weight (kg)										
Initial	34.41	34.43	34.43	34.42	34.45	0.013	-	-	-	-
wk 3	47.76	48.00	47.00	47.75	47.25	0.544	0.87	0.99	0.59	0.86
wk 6	67.85	65.80	65.45	66.34	64.01	0.779	0.25	0.81	0.47	0.60
wk 9	88.45	89.29	89.32	89.35	88.66	0.541	0.64	0.82	0.81	0.79
wk 12	108.41	108.99	108.68	108.56	109.49	0.384	0.63	0.84	0.74	0.52
Average daily gain (g)										
wk 0–3	635.75	645.88	598.50	634.98	609.57	19.129	0.80	0.99	0.45	0.82
wk 3–6	956.46	847.62	878.06	885.04	797.96	26.160	0.13	0.72	0.64	0.33
wk 0–6	796.10	746.93	738.28	760.00	703.77	17.907	0.23	0.80	0.45	0.58
wk 6–9	981.05	1,118.54	1,137.42	1,095.41	1,173.47	24.412	0.01	0.90	0.33	0.55
wk 9–12	950.30	937.94	921.43	914.71	992.44	17.232	0.85	0.56	0.46	0.26
wk 6–12	965.67	1,028.24	1,029.42	1,005.06	1,082.95	14.931	0.06	0.63	0.21	0.23
wk 0–12	880.89	887.59	883.85	882.54	893.36	5.228	0.69	0.87	0.79	0.58
Average daily feed intake (g)										
wk 0–3	1,456.97	1,445.41	1,544.22	1,345.07	1,513.95	33.946	0.95	0.40	0.10	0.65
wk 3–6	2,435.89	2,412.08	2,236.40	2,176.36	2,229.08	56.248	0.25	0.36	0.64	0.39
wk 0–6	1,946.43	1,928.74	1,890.31	1,760.72	1,871.51	38.031	0.41	0.31	0.69	0.41
wk 6–9	3,115.82	3,004.25	2,768.71	3,020.07	2,864.12	54.359	0.14	0.64	0.11	0.74
wk 9–12	3,361.35	2,861.31	3,221.22	3,561.14	3,061.98	84.944	0.32	0.12	0.67	0.02
wk 6–12	3,225.66	2,940.32	2,971.15	3,262.13	2,952.63	56.191	0.15	0.21	0.24	0.16
wk 0–12	2,432.44	2,294.52	2,289.24	2,356.08	2,271.47	36.659	0.20	0.80	0.61	0.65
Gain:feed ratio (G:F ratio)										
wk 0–3	0.436	0.450	0.387	0.476	0.404	0.0145	0.85	0.50	0.05	0.88
wk 3–6	0.398	0.355	0.395	0.410	0.363	0.0152	0.71	0.78	0.96	0.27
wk 0–6	0.413	0.390	0.393	0.433	0.378	0.0119	0.69	0.62	0.35	0.32
wk 6–9	0.316	0.376	0.412	0.363	0.414	0.0121	0.01	0.81	0.07	0.75
wk 9–12	0.286	0.331	0.290	0.257	0.326	0.0104	0.54	0.38	0.51	0.02
wk 6–12	0.301	0.353	0.348	0.309	0.369	0.0094	0.05	0.53	0.15	0.09
wk 0–12	0.363	0.390	0.388	0.375	0.395	0.0061	0.15	0.79	0.52	0.44

SEM, standard error of the mean; BG, β-glucan; VE, vitamin E; SBM, soybean meal.

1) CON, corn-SBM-based diet; LB, corn-SBM-based diet+0.05% β-glucan; LBE, corn-SBM-based diet+0.05% β-glucan+0.02% vitamin E; HB, corn-SBM-based diet+0.1% β-glucan; HBE, corn-SBM-based diet+0.1% β-glucan+0.02% vitamin E.

**Table 6:** Effects of β-glucan with vitamin E supplementation on blood profile and immune response in growing and finishing pigs

Items	Treatment^1)^					SEM	p-value			
	
	CON	LB	LBE	HB	HBE		Diet	BG	VE	BG×VE
Vitamin E (μmol/L)										
Initial	------------------------------------------- 2.23 ------------------------------------					-		-	-	-
wk 3	2.65	2.68	5.53	3.63	5.75	0.396	0.02	0.36	<0.01	0.57
wk 6	3.58	4.85	7.63	4.68	5.88	0.510	0.07	0.36	0.07	0.45
wk 9	4.75	6.45	8.98	6.95	7.93	0.506	0.02	0.78	0.09	0.44
wk 12	5.70	6.85	9.48	5.23	7.58	0.553	0.21	0.13	0.04	0.90
Selenium (μg/L)										
Initial	----------------------------------------- 167.34 ---------------------------------------					-		-	-	-
wk 3	182.00	172.75	175.00	177.00	177.75	2.234	0.30	0.52	0.78	0.89
wk 6	190.75	202.00	186.00	174.75	186.00	3.743	0.69	0.11	0.77	0.11
wk 9	207.50	206.00	187.25	178.25	201.00	5.087	0.26	0.53	0.86	0.08
wk 12	206.75	212.75	209.00	203.50	197.25	5.524	0.94	0.45	0.72	0.93
TNF-α (μg/mL)										
Initial	----------------------------------------- 0.07 -----------------------------------------					-		-	-	-
wk 3	0.09	0.42	0.06	0.02	0.02	0.058	0.76	0.09	0.15	0.15
wk 6	0.13	0.56	0.02	0.10	0.10	0.076	0.70	0.12	0.28	0.10
wk 9	0.46	0.26	0.12	0.07	0.03	0.071	0.07	0.57	0.36	0.75
wk 12	0.34	0.02	0.18	1.05	0.06	0.151	0.99	0.16	0.20	0.09
IL-6 (μg/mL)										
Initial	----------------------------------------- 0.13 ---------------------------------------					-		-	-	-
wk 3	0.11	1.18	0.22	0.02	0.04	0.197	0.58	0.16	0.25	0.31
wk 6	0.24	2.34	0.02	0.10	0.34	0.268	0.99	0.50	0.52	0.27
wk 9	1.22	2.66	0.41	0.07	0.32	0.301	0.10	0.64	0.70	0.88
wk 12	0.96	0.45	0.63	1.05	1.55	0.416	0.73	0.11	0.62	0.49
Lymphocyte (%)										
Initial	----------------------------------------- 52.53 -----------------------------------------					-		-	-	-
wk 3	53.95	61.93	57.00	51.55	50.90	2.859	0.86	0.24	0.69	0.76
wk 6	49.98	56.48	56.02	47.20	52.33	2.151	0.60	0.22	0.65	0.59
wk 9	59.30	64.05	60.15	51.00	51.40	1.750	0.48	<0.01	0.60	0.52
wk 12	60.45	64.03	67.37	56.08	57.93	1.249	0.70	<0.01	0.23	0.72

SEM, standard error of the mean; BG, β-glucan; VE, vitamin E, TNF-α, tumor necrosis factor-α; IL, interleukin; SBM, soybean meal.

1) CON, corn-SBM-based diet; LB, corn-SBM-based diet+0.05% β-glucan; LBE, corn-SBM-based diet+0.05% β-glucan+0.02% vitamin E; HB, corn-SBM-based diet+0.1% β-glucan; HBE, corn-SBM-based diet+0.1% β-glucan+0.02% vitamin E.

**Table 7:** Effects of β-glucan and vitamin E supplementation on pork pH in growing and finishing pigs

Items	Treatment^1)^					SEM	p-value			
	
	CON	LB	LBE	HB	HBE		Diet	BG	VE	BG×VE
Time after slaughter										
0 h	5.75	5.74	5.62	5.65	5.68	0.026	0.27	0.78	0.48	0.22
3 h	5.72	5.49	5.64	5.43	5.52	0.031	<0.01	0.11	0.03	0.58
6 h	5.36	5.44	5.55	5.46	5.46	0.030	0.18	0.63	0.44	0.47
12 h	5.54	5.46	5.58	5.51	5.52	0.022	0.74	0.90	0.23	0.31
24 h	5.32	5.41	5.49	5.44	5.34	0.020	0.03	0.14	0.71	0.03

SEM, standard error of the mean; BG, β-glucan; VE, vitamin E; SBM, soybean meal.

1) CON, corn-SBM-based diet; LB, corn-SBM-based diet+0.05% β-glucan; LBE, corn-SBM-based diet+0.05% β-glucan+0.02% vitamin E; HB, corn-SBM-based diet+0.1% β-glucan; HBE, corn-SBM-based diet+0.1% β-glucan+0.02% vitamin E.

**Table 8:** Effects of β-glucan and vitamin E supplementation on pork color in growing and finishing pigs

Items	Treatment^1)^					SEM	p-value			
	
	CON	LB	LBE	HB	HBE		Diet	BG	VE	BG×VE
CIE value^2)^, L*										
0 h	49.76	47.30	43.87	36.70	43.50	1.618	0.14	0.28	0.96	0.32
3 h	41.87	46.79	45.58	44.87	49.09	1.182	0.13	0.77	0.58	0.32
6 h	45.20	47.32	42.72	39.04	41.77	1.620	0.56	0.23	0.80	0.34
12 h	44.16	47.84	42.37	46.16	44.16	1.068	0.73	0.98	0.15	0.49
24 h	44.27	53.07	50.06	46.82	41.45	1.709	0.39	0.06	0.26	0.75
CIE value^2)^, a*										
0 h	2.50	2.97	3.52	3.19	3.57	0.141	0.12	0.64	0.12	0.75
3 h	2.74	3.11	2.60	3.46	2.70	0.209	0.68	0.65	0.21	0.80
6 h	3.35	2.42	3.45	2.95	3.44	0.207	0.60	0.58	0.12	0.57
12 h	2.89	3.35	2.54	2.65	3.77	0.259	0.78	0.66	0.80	0.13
24 h	3.58	1.97	2.22	2.58	4.00	0.361	0.33	0.15	0.31	0.46
CIE value^2)^, b*										
0 h	8.88	8.80	8.76	7.86	9.30	0.445	0.87	0.86	0.53	0.51
3 h	10.04	9.18	9.19	8.87	8.04	0.346	0.19	0.37	0.61	0.60
6 h	10.80	9.54	9.78	9.41	9.08	0.389	0.21	0.67	0.96	0.76
12 h	11.09	9.41	10.23	8.54	9.66	0.396	0.12	0.42	0.28	0.86
24 h	8.55	8.75	8.83	9.34	9.51	0.335	0.55	0.45	0.88	0.96

SEM, standard error of the mean; BG, β-glucan; VE, vitamin E; SBM, soybean meal.

1) CON, corn-SBM-based diet; LB, corn-SBM-based diet+0.05% β-glucan; LBE, corn-SBM-based diet+0.05% β-glucan+0.02% vitamin E; HB, corn-SBM-based diet+0.1% β-glucan; HBE, corn-SBM-based diet+0.1% β-glucan+0.02% vitamin E.

2) CIE L*, luminance or brightness (varies from black to white); CIE a*, red-green component (+a = red, −a = green); CIE b*, yellow-blue component (+b = yellow, −b = blue).

**Table 9:** Effects of β-glucan and vitamin E supplementation on carcass traits and physiochemical properties in growing and finishing pigs

Items	Treatment^1)^					SEM	p-value			
	
	CON	LB	LBE	HB	HBE		Diet	BG	VE	BG×VE
Carcass traits										
Carcass weight (kg)	85.25	86.50	88.00	84.00	86.00	0.478	-	-	-	-
Carcass yield (%)	77.33	77.06	77.36	76.71	76.96	0.090	-	-	-	-
Grade^2)^	1.50	2.25	1.50	1.25	1.50	0.168	0.77	0.20	0.52	0.20
Backfat thickness (mm)	23.50	24.50	24.00	21.75	22.75	0.952	0.92	0.40	0.92	0.75
Physiochemical properties										
Cooking loss (%)	20.52	22.70	21.40	22.63	19.36	0.463	0.33	0.26	0.12	0.29
Shear force (kg/0.5 inch^2^)	30.13	38.25	30.16	35.20	31.98	2.007	0.49	0.90	0.25	0.61
WHC	72.15	68.97	68.54	67.29	70.65	0.984	0.22	0.93	0.53	0.42

SEM, standard error of the mean; BG, β-glucan; VE, vitamin E; WHC, water holding capacity; SBM, soybean meal.

1) CON, corn-SBM-based diet; LB, corn-SBM-based diet+0.05% β-glucan; LBE, corn-SBM-based diet+0.05% β-glucan+0.02% vitamin E; HB, corn-SBM-based diet+0.1% β-glucan; HBE, corn-SBM-based diet+ 0.1% β-glucan+ 0.02% vitamin E.

2) Grade: grade 1+ = 1; grade 1 = 2; grade 2 = 3.

**Table 10:** Effects of β-glucan and vitamin E supplementation on inosine monophosphate in growing and finishing pigs

Items	Treatment^1)^					SEM	p-value			
	
	CON	LB	LBE	HB	HBE		Diet	BG	VE	BG×VE
IMP (mg/kg)	1,097.5	1,420.8	1,787.1	1,947.8	1,411.8	103.02	0.02	0.70	0.67	0.03

SEM, standard error of the mean; BG, β-glucan; VE, vitamin E; IMP, inosine monophosphate; SBM, soybean meal.

1) CON, corn-SBM-based diet; LB, corn-SBM-based diet+0.05% β-glucan; LBE, corn-SBM-based diet+0.05% β-glucan+0.02% vitamin E; HB, corn-SBM-based diet+0.1% β-glucan; HBE, corn-SBM-based diet+0.1% β-glucan+0.02% vitamin E.

**Table 11:** Effects of β-glucan and vitamin E supplementation on economic benefit in growing and finishing pigs

Items	Treatment^1)^					SEM	p-value			
	
	CON	LB	LBE	HB	HBE		Diet	BG	VE	BG×VE
Feed cost per weight gain (won/kg)										
wk 0–3	1,199	1,174	1,351	1,131	1,305	39.2	0.31	0.79	0.06	0.99
wk 3–6	1,349	1,529	1,333	1,290	1,468	57.3	0.70	0.86	0.95	0.18
wk 6–9	1,610	1,349	1,216	1,384	1,231	47.2	0.02	0.10	0.12	0.91
wk 9–12	1,699	1,585	1,625	1,803	1,486	47.1	0.33	0.92	0.19	0.10
wk 0–12	961	900	902	929	888	13.2	0.26	0.45	0.52	0.48
Total feed cost per pig (won/head)										
wk 0–3	15,910	15,783	16,862	14,688	16,523	370.7	0.39	0.57	0.10	0.65
wk 3–6	26,600	26,339	24,421	23,765	24,341	614.2	0.52	0.44	0.63	0.38
wk 6–9	32,716	31,544	29,071	31,710	30,073	570.7	0.26	0.70	0.11	0.74
wk 9–12	38,869	30,873	31,197	34,252	31,002	589.9	0.04	0.31	0.24	0.16
wk 0–12	104,205	98,297	98,071	100,934	97,309	1,570.4	0.46	0.59	0.61	0.65
Days to market weight (reached at 115 kg body weight, d)										
	107.7	107.5	106.7	105.3	104.0	0.58	0.21	0.11	0.42	0.84

SEM, standard error of the mean; BG, β-glucan; VE, vitamin E; SBM, soybean meal.

1) CON, corn-SBM-based diet; LB, corn-SBM-based diet+0.05% β-glucan; LBE, corn-SBM-based diet+0.05% β-glucan+0.02% vitamin E; HB, corn-SBM-based diet+0.1% β-glucan; HBE, corn-SBM-based diet+0.1% β-glucan+0.02% vitamin E.
